# The complete chloroplast genome of Yishui Lily 140 (Liliaceae *Lilium lancifolium*) yielded by next-generation sequencing

**DOI:** 10.1080/23802359.2021.1996292

**Published:** 2021-11-03

**Authors:** Huan-Yu Wu, Jin-Hai Peng, Pei-Feng Ding, Feng-Gang Zhang, Xi-Xiu Zhang, Xiang-Yan Chen, Qi-Chao Zhao, Lin Wang, Liang Wang, Yan Wang, Yun-Guo Liu

**Affiliations:** a College of Life Sciences and Technology, Xinjiang University, Urumqi, P. R. China; b College of Life Sciences, Linyi University, Linyi, P. R. China; c Linyi Academy of Agricultural Sciences, Linyi, P. R. China; d Agriculture Comprehensive Service Center of Taiping Neighbourhood, Linyi, P. R. China; e Agriculture Comprehensive Service Center of Baishabu Town, Linyi, P. R. China; f Forestry Development Center of Pingyi County, Pingyi, P. R. China; g Agricultural and Rural Bureau of Lanling County, Lanling, P. R. China

**Keywords:** Yishui Lily 140, *Lilium lancifolium* (*L. lancifolium*), chloroplast (cp) genome

## Abstract

Yishui Lily 140 (*Lilium lancifolium*) is a hybrid lily species which was bred from wild lily varieties due to its edible and medicinal value. In this study, we have sequenced the complete chloroplast (cp) of *L. lancifolium*. The complete cp sequence is 152,643 bp long, with a large single copy (LSC) region of 82,084 bp, a small single copy (SSC) region of 17,513 bp, and two inverted repeat (IR) regions of 26,492 bp each. The GC contents of the complete cp genomes are 37.0%. It contains 132 genes, including 86 coding genes, 8 ribosomal *RNAs*, and 38 transfer *RNAs*. Among them, 16 different genes have a single intron and the remaining two genes have double introns, including nine cis-splicing and one trans-splicing genes. Compared with other species, we found three high variation hot spots and 96 repeats sequence. The genetic information of *Lilium* can be enriched as well as identifying proximal species. They are edible and have medicinal value for humans. Therefore, sequencing of Yishui Lily 140 is important to explore the cp genome composition.

Lily is a valuable plant with more than 110 species mainly distributed throughout East Asia and North America (Liang and Tanmuram 2000; Qi et al. 2020). Not only is the lily showy in color, but also its bulb has edible and medicinal value for the treating of various long-term disease (Ma et al. 2017).

Commercial lily breeding is mainly derived from Asiatic hybrids, Oriental hybrids, and *Longiflorum* hybrids (Cao et al. 2016). Yishui Lily 140 (*L. lancifolium*) was selected and bred from wild and traditional lily in the Research and Development (R&D) center of Edible Lily in Yishui, Shandong Province. It is the polyploid complex which was backcrossed by the Asiatic hybrids and distant hybrids generation of *Longiflorum* hybrids and Asiatic hybrids. Yishui Lily 140 completely changes the weakness of poor adaptability and long growth cycle of traditional lily, and can be planted at any time from April to August every year (Cai and Jia 2005). In China, *L. lancifolium* is extensively used as spice, food as well as traditional Chinese medicinal material. Yishui edible lily tastes delicious, especially the petals can be directly eaten without bitter taste (Xu et al. 2016). As more and more edible lily varieties are being developed by hybrid, in the future, more complete cp sequences become available. Based on the complete sequencing of cp genome, the genetic background can be better understood which is helpful in promoting the development of edible lilium in Yishui, and can also enrich the genetic information for further researches of *L. lancifolium*.

The total genomic DNA was extracted from fresh leaves of Yishui Lily 140 (Shi et al. 2012) planted in Yishui County, Linyi City, Shandong Province (N: 35°47′26′′, E: 118°37′47′′), China in July, 2020. Additional specimens were kept in the College of Life Sciences, Linyi University, Linyi, China. A genomic library consisting of an insert size of 500 bp was constructed using NEB DNA Library Prep Kit (Illumina, San Diego, CA, USA), The complete cp genome of Yishui Lily 140 was sequenced by next-generation sequencing system MGISEQ-2000 (PE 150) and 6 Gb raw data was generated.

After data quality control, adapter and small segments with length less than 50 bp were removed, and the clean date was further assembled using NOVO Plasty (Dierckxsens et al. 2017). Annotations of the complete cp genome were conducted by the software GBseq based on previously published cp complete genome of *Lilium* (KY748297.1) (Du et al. 2017). The complete cp genome sequences were aligned using MAFFT (Katoh et al.^2002^). Then schematic representation of the complete cp genome was drawn in CPGAVAS2 (http://www.herbalgenomics.org/cpgavas2/) (Shi et al. 2019). The complete cp genome sequence and annotation results have been submitted to the NCBI (MW465411).

The complete cp genome of Yishui Lily 140 is 152,643 bp in length, displaying a typical quadripartite structure consisted of four subregions: a pair of inverted repeats (IR) regions (52,984 bp), a large single copy (LSC) region (82,083 bp) and a small single copy (SSC) region (17,576 bp). The GC contents of LSC, SSC, and IR are 34.82, 30.74, and 42.48%, respectively, and the total GC contents of the complete cp genome are 37.0%. In the complete cp genome, 132 genes are reported, including 86 protein-coding genes, eight ribosomal *RNA* genes, and 38 transfer *RNA* genes. The genes involved in the synthesis of photosynthesis are grouped into six major groups, such as ATP synthase, photosystem I, photosystem II, NADH-dehydrogenase, cytochrome b/f complex, and rubisco. Among the 132 genes, 16 different genes (*atp*F, *ndh*A, *ndh*B, *rrn*23, *rpo*C1, *rpl*16, *rps*12, *rps*16, *trn*A-UGC, *trn*G-GCC, *trn*I-GAU, *trn*K-UUU, *trn*L-UAA, *trn*V-UAC, *pet*B, and *pet*D) have a single intron and only *paf*I and *clp*P1 genes contain double introns. In Yishui Lily 140, 9 cis-clipping genes including *paf*I, *clp*P1, *rps*16, *atp*F, *prt*B, *pet*D, *rpl*16, *ndh*B and *ndh*A, and a trans-splicing gene *rps*12 were detected by CPGview-RSG (http://www.herbalgenomics.org/cpgview/). A total of 41 direct repeats, 45 tandem repeats and 10 palindromic repeats have been identified in the cp genome of Yishui Lily 140 by the CPGAVAS2.

For phylogenetic analysis, 14 *Lilium* species and one outgroup species were selected to construct the phylogenetic tree constructed with 1000 bootstrap replicates by the MEGA version 7 (Philadelphia, USA) for the chloroplast genome of 13 *Lilium* species, one outgroup species downloaded from NCBI GenBank and the Yishui Lily 140 to confirm the phylogenetic position (Kumar et al. 2016). This neighbor-joining tree (N–J tree) is composed mostly of nine nodes with support values of 100% and two nodes with support values greater than 90%.

The N-J tree also shows that 14 *Lilium* species are grouped into two branches and the Yishui Lily 140 is closely related to three *L. Lancifolium* (KY748297.1, KY940844.1, and MH177880.1) (Figure 1). And from the sequence alignment analysis results of these four sequences on mVISTA (http://genome.lbl.gov/vista/mvista/submit.shtml), we find that there are some high variation hot spots. Two of them are located in conserved non-coding sequence (CNS) of *trn*T-UGU gene and the fragment between *pet*A and *psb*J gene. Another one is located in the untranslated region of *ccs*A gene.

**Figure 1.**
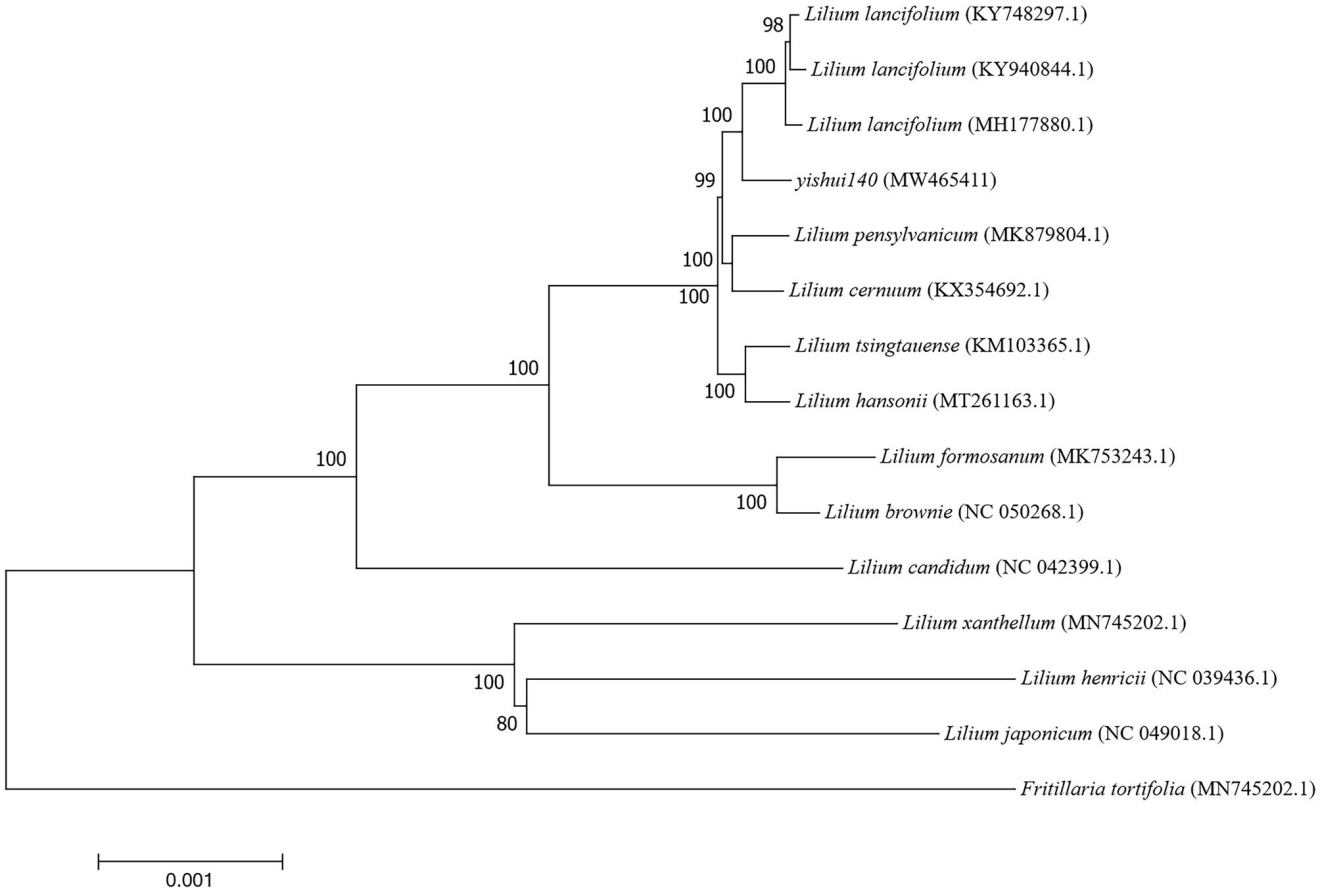
Neighbor-joining tree based on combining the complete cp genome sequences of 14 *Lilium* species and one outgroup species by using MEGA version 7. Bootstrap values based on 1000 replicates are shown at branch nodes.

## Data Availability

The genome sequence data that support the findings of this study are openly available in GenBank of NCBI at (https://www.ncbi.nlm.nih.gov/) under the accession no. MW465411. The associated BioProject, SRA, and Bio-Sample numbers are PRJNA753155, SRR15403610, and SAMN20691378, respectively.
